# Non-penetrating deep sclerectomy with the sub flap (Ahmed’s) suture: a 12-month comparative study

**DOI:** 10.1038/s41433-022-02102-6

**Published:** 2022-05-31

**Authors:** Ahmed Mostafa Abdelrahman, Lameece Moustafa Hassan, Mina Maged Habib

**Affiliations:** 1 Professor of Ophthalmology, Cairo University, Cairo, Egypt; 2 Lecturer of Ophthalmology, Cairo University, Cairo, Egypt; 3 Assistant lecturer of Ophthalmology, Cairo University, Cairo, Egypt

**Keywords:** Surgery, Glaucoma

## Abstract

**Purpose:**

To assess the IOP-lowering effect of adding a mattress suture (Ahmed’s suture) to non-penetrating deep sclerectomy (NPDS), in patients with open angle glaucoma over a 12-month follow-up period.

**Methods:**

This is a randomized controlled study comparing 52 eyes with a sub-flap Ahmed’s suture modified NPDS (group A) and 51 with a conventional NPDS (group B). Success of surgery was categorized as complete success if the IOP remained between 6 and 18 mmHg without medications and as qualified if topical medications were required.

**Results:**

The post-operative IOP at the 1st week, 3rd, 6th, 9th & 12th months follow ups in group A were significantly lower (7.3 ± 2.1, 12.0 ± 2.3, 12.6 ± 2.7, 13.6 ± 3.4 & 13.8 ± 3.8 mmHg) than in B (9.2 ± 1.9, 14.0 ± 3.1, 14.8 ± 2.9, 15.4 ± 2.6 & 15.7 ± 2.7 mmHg) (*p* = 0.001, *p* = 0.001, *p* = 0.002, *p* = 0.027 & *p* = 0.029 respectively). The percentage of IOP reduction after 1 year was significantly higher in group A than in group B (49% vs. 36.5%). At the end of the 12-month follow-up, 81% of group A and 69% of group B were considered as complete success. Multivariate regression analysis showed lower 1st week post-operative IOP was associated with better outcome.

**Conclusion:**

In conclusion, the Ahmed’s suture, a simple, novel and economic modification, maintains lower IOP levels and has a higher success rate over conventional DS, as it is 30% more effective in reducing the IOP.

## Introduction

Non-Penetrating Deep sclerectomy (NPDS) was first described in 1990, with the intention of averting the often-catastrophic complications of penetrating surgery: hypotony, hyphema, lost anterior chamber, choroidal detachment, effusion or haemorrhage and endophthalmitis [1, 2]. It thus became an established technique, where IOP reduction was achieved by augmenting the natural route of aqueous drainage. The idea is to remove a flap from the depth of the sclera where both the juxacanalicular trabecular meshwork and the inner wall of Schlemm canal (SC) (these two structures pose the highest resistance to outflow of aqueous). This allows percolation of aqueous humor through a thin trabeculo-descemet membrane (TDM), then accumulate in a space within the sclera known as “the decompression space,” and finally be drained through the subconjunctival space [3–5].

Since its description a significant concern remained in NPDS: how to keep the decompression space open; as anti-metabolites alone might not sufficiently achieve this goal. Thus, multiple modifications were implemented, including the insertion of numerous types of implant materials under the scleral flap in order to retain the “decompression space”, such as the collagen derived Aquaflow implants, reticulated hyaluronic acid or even autologous scleral implants [6].

“The sub flap Ahmed’s mattress suture” is a novel, simple and economical technique in which 10/0 nylon suture in mattress orientation is sutured under and reaching beyond the edges of the superficial scleral flap. These “sub-flap mattress sutures” act as a hinge maintaining the decompression space and thus augmenting the intraocular pressure (IOP)-lowering effect of NPDS. The nylon suture is nonabsorbable and convenient. Our initial 3-month study found that use of this suture maintained lower IOP levels and with lower rates of required of additional YAG- goniopuncture than in the control group, where a conventional NPDS was performed [7].

This study compares deep sclerectomy (DS) patients, with an additional Ahmed’s suture, with regards to IOP changes throughout follow-up, the need for any additional intervention and the occurrence of any complications, to those with a conventional DS over a period of 9 months.

## Materials and methods

This study is a prospective randomized control trial comparing the outcome of modified DS vs. that conventional DS over a period of 12 months.

It includes 105 eyes from 70 patients with open angle glaucoma who were not controlled despite being on full medical treatment. Patients were enlisted from the Giza Specialized eye Center; a tertiary Glaucoma care facility. In this study we excluded those with previous intervention for glaucoma, including laser therapy, or even lens extraction. Patients with prior refractive surgery were also excluded.

Approval for the study was obtained from the ethics committee of the Giza Specialized Eye Center, Egypt. The study adheres to tenets of the Declaration of Helsinki. Before the intervention all enlisted patients were counselled, and all patients signed an informed consent.

In this study eyes were randomized into two groups using excel created random numbers (allocated on the day of surgery): those with a sub- flap mattress suture (Ahmed’s suture) modified NPDS (group A) and those with a conventional NPDS (group B).

### Pre-operative assessment

A full history was taken from all participants, followed by a complete ophthalmological examination including: slit-lamp anterior segment assessment, IOP measurement using the Goldmann’s applanation tonometry, gonioscopy (confirm open angles) and dilated fundus examination.

Diagnosis of glaucoma was established using perimetry and serial optical coherence tomography of optic nerve head (OCT ONH) to assess retinal nerve fibre layer thickness changes prior to surgery. Central corneal thickness (CCT) was also measured by pachymetry/topography.

### Surgical technique

Every patient signed an informed consent. Surgery was performed under complete aseptic conditions and the periocular skin was washed with Betadine 10%^®^ and draped. Diluted Betadine eye drops 5%^®^ were instilled before starting the surgery.

All the interventions were performed by the one glaucoma surgeon (A.M.A.) and using peribulbar anaesthesia.

In the first group (standard NPDS), a 7/0 Vicryl corneal traction suture was placed in the superior cornea to increase exposure of the superior conjunctiva. Next, a 10 mm fornix-based conjunctival flap was dissected. Any bleeding point was stopped by minimal diathermy. The superficial scleral flap (4 × 3 mm) was fashioned by crescent blade and its dissection was continued forward allowing exposure of the upper two mm of clear cornea. Microsponges were soaked with Mitomycin-C (MMC)^®^ (prepared immediately prior to surgery at a concentration of 0.4 mg/mL) were placed below the scleral flap, the subconjunctival space and moved upwards toward the upper fornix. The duration of application of the sponges was 2 min, following the entire area was well irrigated with a 30 mL balanced salt saline solution. Using a microvitreoretinal blade a paracentesis was made and intracameral acetylcholine was injected to constrict the pupil.

Close to the edges of the superficial scleral flap, a deep scleral flap was dissected, starting just inside, and continued in order to unroof the SC and expose the TDM. Percolation was assessed with a cellulose microsponge, which was also used to detach any trabecular meshwork cells. With a fine-tipped forceps the floor of the Schlemm’s canal was carefully peeled. Followed by excision of the deep scleral flap. The superficial flap was repositioned without sutures. Using two 10/0 Nylon sutures the conjunctival was closed and assessed for leakage with a dry cellulose sponge. Gentamicin and dexamethasone solutions were used to irrigate the surface of the conjunctiva, and finally a steroid-antibiotic ointment was spread over the ocular surface.

In the mattress suture modified NPDS group, the procedure was identical to standard surgery up to excision of the deep scleral flap, then the sub flap mattress Ahmed’s suture was placed.

### The sub-flap mattress Ahmed’s suture

**Fig. 1:**
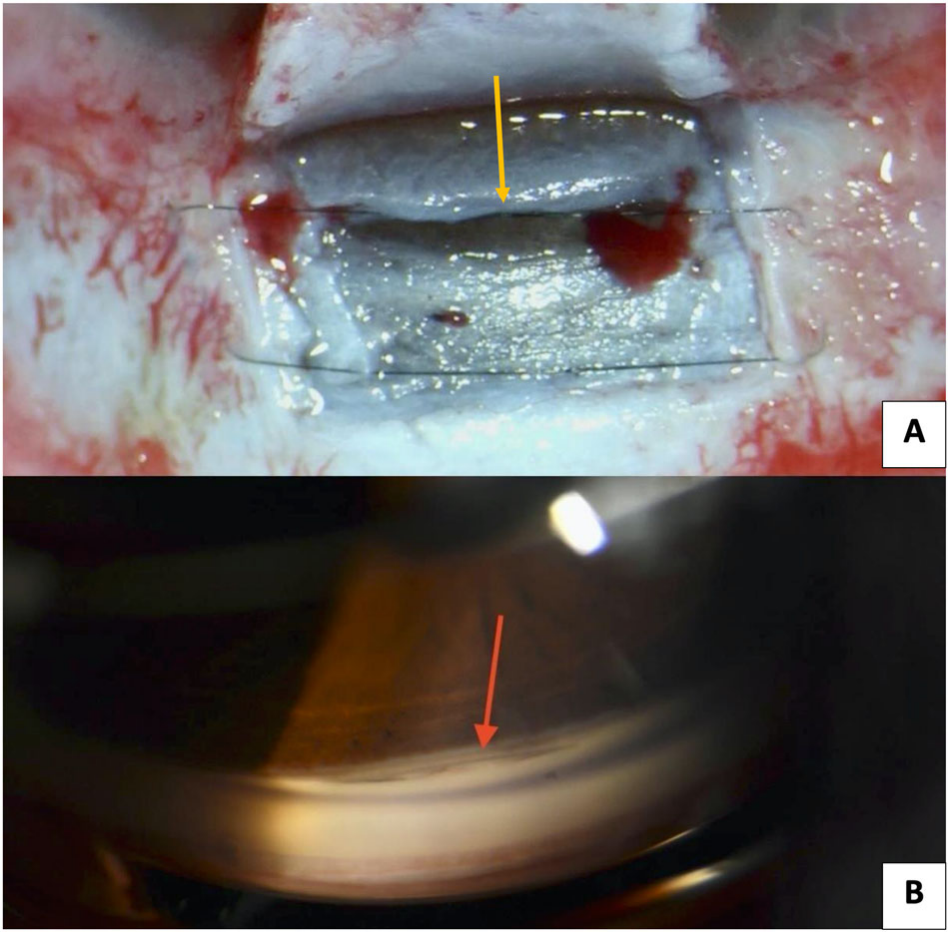
Ahmed’s suture. **A** The sub-flap mattress 10/0 Nylon suture placed under the superficial scleral flap and extending beyond the edges of the flap. The yellow arrow points to the proximal limb of the suture lying on the Trabeculo-Descemet’s membrane. **B** Gonioscopic appearance of the surgical site with the 10/0 Nylon suture extending horizontally. This suture can be used as a land mark during YAG-goniopuncture *(Bottom)*.

A 10/0 nylon suture (Alcon)^®^ mattress suture was secured under the superficial flap and extended 1 mm beyond its edges. The technique of suture placement used was the same as first described by *Abdel Rahman and Habib* [7] (Fig. 1).

Postoperative treatment consisted of topical prednisolone acetate ophthalmic suspension (Pred Forte 1%) and gatifloxacin (Tymer 0.3%) antibiotic drops. The drops were used five times per day for 2 weeks with gradual tapering over 1 month.

### Follow up

Follow up was conducted by a masked second glaucoma consultant (M.M.H.) for 12 months with repeated assessment including bleb evaluation and IOP measurement.

The patients were closely followed up for 12 months (including at least 6 post-operative visits: at 1 week, 1 month, 3 months, 6 months, 9 months & 12 months). Each visit included an anterior segment examination with bleb assessment and monitoring for the development of visually significant cataracts, IOP measurement using Goldmann’s applanation tonometry after staining by sterile fluorescein strips, fundus examination for choroidal detachments and reporting any patient’s complaints. The conjunctival sutures were removed on the slit lamp after 3–4 weeks if a foreign body sensation was reported by the patients or if discharge was found on the sutures.

Success of surgery was categorized as complete success if the IOP remained between 6 and 18 mmHg without any medication throughout the12 month follow up period and as qualified success with the same IOP, but with the addition of topical medications. On the other hand, failure was deemed if any of the aforementioned success criteria was not met without the need for another major glaucoma surgery or the occurrence of any sight-threatening complication [8].

### Statistical methods

Data were statistically described in terms of mean ± standard deviation (±SD), median and range, or frequencies (number of cases) and percentages when appropriate. Comparison of normally distributed numerical variables between the two study groups was done using the student *t* test. A bar chart was used to show the changes in IOP over time in both groups. A probability value (*p* value) < 0.05 is considered statistically significant. All statistical calculations will be done using computer programs Microsoft Excel 2013 (Microsoft Corporation, NY, USA).

Sample size calculation was done using the comparison of the change in IOP between patients treated with modification to standard NPDS by adding a sub-flap mattress suture after excision of the deep scleral flap and those treated with the standard technique. As determined in the pilot study, *(Abdelrahman and Habib)* [7], the mean ± SD of IOP change in modified technique group was ~52.9 ± 13.1%, while in the standard technique group it was ~40.5 ± 10.3. Accordingly, the minimum sample size needed to reject the null hypothesis with 80% power at α = 0.05 level using Student’s *t* test for independent samples was 19 participants in each group. Sample size calculation was done using PS power and sample size calculations software, version 3.0.11 for MS Windows (William D. Dupont and Walton D., Vanderbilt University, Nashville, Tennessee, USA).

Multivariate linear and logistic regression analysis models were used to test for the preferential effect of the important risk factors on 1-year post-operative IOP, postoperative number of medications, and the need for additional intervention. Two-sided *p* values < 0.05 was considered statistically significant. All statistical calculations were done using computer program IBM SPSS (Statistical Package for the Social Science; IBM Corp, Armonk, NY, USA) release 22 for Microsoft Windows.

## Results

This study included 52 eyes in group A and 51 eyes in group B (as one eye was eliminated in each group due to intraoperative conversion to penetrating surgery).

The demographics of the patients in each group were similar, with no statistically significant difference when comparing age, gender, pre-operative IOP and the number of pre-operative anti-glaucoma medications used (Table 1). All the patients in each group were classified as either POAG or JOAG, and two patients had pigmentary OAG in each group.

**Table 1:** Patient Demographics.

	Modified NPDS	Standard NPDS	*P* value
No. of eyes	52	51	
No. of patients	36	33	
Gender (M/F)	24/12	23/10	
No. of POAG	15/36	14/33	
No. of JOAG	19/36	17/33	
No. of pigmentary OAG	2/36	2/33	
Age (years)	38.1 ± 13.3	38.4 ± 15.0	0.93
Pre-op IOP (mm Hg)	27.4 ± 6.3	27.1 ± 6.6	0.78
Pre-op Meds	3.4 ± 1.0	3.5 ± 1.2	0.67

Modified NPDS, Modified non-penetrating deep sclerectomy using mattress suture.

Standards NPDS, Standard technique of non-penetrating deep sclerectomy.

Pre-op Meds, Pre-operative antiglaucoma medications.

*POAG* Primary open angle glaucoma, *JOAG* Juvenile open angle glaucoma, *M/F* Male/Females, *IOP* Intra-ocular pressure.

**Fig. 2:**
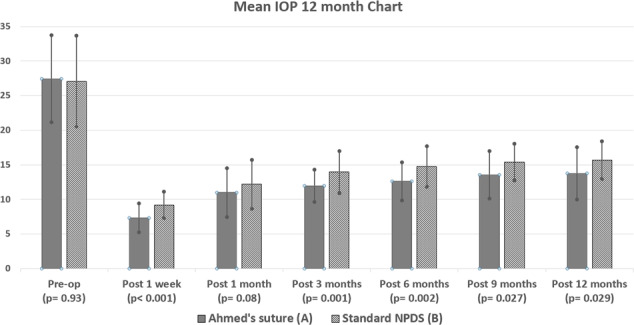
Mean IOP throughout the 12 month follow-up period. Bar Chart comparing IOP changes in both groups throughout the 12 month follow-up period.

The post-operative IOP at the 1st week, the 3rd, the 6th, 9th, & 12th months follow ups was statistically significantly lower in group A (7.3 ± 2.1, 12.0 ± 2.3, 12.6 ± 2.7, 13.6 ± 3.4 & 13.8 ± 3.8 mmHg) than in B (9.2 ± 1.9, 14.0 ± 3.1, 14.8 ± 2.9, 15.4 ± 2.6 & 15.7 ± 2.7 mmHg) (*p* = 0.001, *p* = 0.001, *p* = 0.002, *p* = 0.027 & *p* = 0.029 respectively) (Fig. 2) (Table 2).

**Table 2:** Post- operative follow-up data in both groups over the 12-month study period.

	Modified DS	Standard DS	*P* value
IOP 1 week (mm Hg)	7.3 ± 2.1	9.2 ± 1.9	0.001
IOP 1 mo. (mm Hg)	11.0 ± 3.5	12.2 ± 3.5	0.086
IOP 3 mo. (mm Hg)	12.0 ± 2.3	14.0 ± 3.1	0.001
IOP 6 mo. (mm Hg)	12.6 ± 2.7	14.8 ± 2.9	0.002
IOP 9 mo. (mm Hg)	13.6 ± 3.4	15.4 ± 2.6	0.027
IOP 1 yr. (mm Hg)	13.8 ± 3.8	15.7 ± 2.7	0.029
IOP drop 3 mo. (mm Hg)	15.5 ± 5.9	12.5 ± 6.4	0.017
IOP drop 6 mo. (mm Hg)	14.7 ± 6.6	10.8 ± 5.9	0.009
IOP drop 9 mo. (mm Hg)	14.5 ± 6.3	10.4 ± 6.5	0.027
IOP drop 1 yr. (mm Hg)	14.3 ± 6.6	10.3 ± 6.9	0.041
% Drop IOP at 3 months	54.9% ± 10.6	45.0% ± 15.6	0.001
% Drop IOP at 6 months	51.7% ± 12.6	40.3% ± 15.2	0.001
% Drop IOP at 9 months	50.1% ± 11.4	37.6% ± 15.4	0.003
% Drop IOP at 1 yr	49.0% ± 14.5	36.5% ± 16.6	0.006
Post Op meds	0.2 ± 0.3	0.5 ± 0.8	0.046
Goniopuncture	6/52	9/51	
Needling	2/52	6/51	
Trauma & iris prolapse	2/52	2/51	
Complete success	42/52	35/51	
Qualified success	9/52	15/51	

Modified NPDS, Modified non-penetrating deep sclerectomy using mattress suture.

Standards NPDS, Standard technique of non-penetrating deep sclerectomy.

Complete success, IOP of 6–18 mmHg with no medications.

Qualified success, IOP of 6–18 with topical medications.

*Mo* months, *Post-op Meds* Post-operative antiglaucoma medications, *IOP* Intra-ocular pressure.

The reduction in IOP after 3, 6, 9 & 12 months was significantly larger in group A than in B (*p* = 0.017, *p* = 0.009, *p* = 0.027 and *p* = 0.041) (Table 2).

The percentage of reduction in IOP after 3, 6, 9 & 12 months when compared to the pre-operative IOP in the 2 groups was highly statistically significant (*p* = 0.001, *p* = 0.001, *p* = 0.003, and *p* = 0.006) (Table 2). Thus, adding Ahmed’s suture to NPDS made the surgery ~30% more effective.

On comparing the post-operative IOP at 3 & 6 months, at 6 & 9 months and at 9 & 12 months in each group individually, no statistically significant difference was found either in group A or group B reflecting the stability of the post-operative course in both groups.

Both goniopuncture and needling were performed at a higher rate in group B than in group A (12% and 4% respectively in group A as compared to 18% and 12% in group B). Regarding post-operative antiglaucoma medications the average was 0.2 meds in group A vs. 0.5 in group B, which was statistically significant (*p* = 0.046) (Table 2).

No patients developed significant cataracts over the 1 year follow up period. Complications were negligible in both groups, however, coincidently during the follow up period 2 eyes in group A and 1 in group B were subjected to a blunt trauma resulting in scleral dehiscence with iris prolapse, which needed surgical iridectomy and repair of the sclera and conjunctiva.

In group A, a larger number of eyes, 42/52 (81%) were considered as complete success while 9/52 as a qualified success (17%). While, in group B, 35/51 (69%) were considered complete success and 15/51 (29%) eyes as a qualified success (Table 2).

Multivariate linear and logistic regression analysis showed that higher pre-operative IOP was associated with the need for increased post-operative medications (*p* = 0.029) and further interventions (goniopuncture and needling) (*p* = 0.021). The other significant risk factor was higher post-operative IOP at the first week, which according to regression analysis was associated with higher IOP at 12 months follow up (*p* = 0.002) and with increased post-operative antiglaucoma medications. (*p* = 0.026) (Table 3).

**Table 3:** Multivariate regression analysis of risk factors over the 12-month study period.

	Post-operative IOP		Post-operative medications		Additional interventions	
	*t*	*p* value	*t*	*p* value	Wald	*p* value
Age	−0.864	0.391	0.703	0.484	0.711	0.399
Gender	0.785	0.436	−0.408	0.684	0.161	0.688
Pre-op IOP	0.650	0.518	**2.210**	**0.029**	**5.327**	**0.021**
Pre-op meds	0.977	0.333	1.720	0.088	0.478	0.489
Post-op IOP 1 week	**2.295**	**0.026**	**3.200**	**0.002**	0.738	0.390

Statistically significant risk factors are shown in bold.

## Discussion

The Sub Flap Mattress (Ahmed’s) Suture was first introduced in 2020, by *Abdelrahman and Habib* and the initial 3-month study showed very promising results with greater IOP drop and fewer needed additional goniopuncture that in the conventional DS. [7]

Likewise, this study, which continued its comparative trial using the Ahmed’s suture over 12 months, showed significantly lower IOP measurements in almost all follow-up visits, greater IOP drops and finally an almost 30% higher efficiency rate.

Lower early post- operative IOP was reported to be a good prognostic indicator, with higher long term success in both sub scleral trabeculectomy (SST) and DS [9, 10]. Okimoto et al., found a lower IOP under 8 mm Hg at 2 weeks after SST was found to be associated with maintaining the postoperative IOP lower than 11–15 mm Hg over a prolonged period [9]. Shaarawy et al., on analysis of 105 patients found that IOP in the first postoperative day could be utilized as a prognostic factor in DS. Lower IOP in the first postoperative day could be correlated with higher success probabilities [10].

In our study the addition of the Ahmed’s suture provided a significantly lower early IOP, 7.3 ± 2.1 in Group A vs. 9.2 ± 1.9 in group B (*p* = 0.001) in the 1st follow-up visit (1-week postoperative). Multivariate regression analysis of risk factors showed that lower IOP at the 1st week follow up visit is associated with lower IOP at 12 months follow up and fewer antiglaucoma medications usage. Thus, the modified technique in group A led to a lower initial post-operative IOP with a better prognosis and higher success rate at 12 months.

Numerous modifications have been made to improve the outcomes of DS. The concept behind using collagen or autologous scleral implant is to enhance the filtration by maintaining the patency of the ‘subscleral aqueous decompression space’ [11]. Use of a lyophilized porcine scleral collagen implant after 96 months, 91% of patients achieved qualified success (IOP below 21 mm Hg with or without the use of antiglaucoma drugs) and 57% achieved complete success (IOP below 21 mm Hg without medication). A Nd-YAG goniopuncture was required in 54 patients (51%) [12].

However, the use of collagen implants is not only expensive and not always readily available but often leads to fibrosis and thus the high rate of needed goniopuncture. Autologous scleral implants attempted to overcome these drawbacks and in a 12-month follow-up, “complete success” was achieved in 50% of cases (IOP l<18 mmHg without medication) and “qualified success” in 85% of cases (IOP <18 mmHg with medication). An additional Nd:YAG goniopuncture was required in 45% of the participants. However, the authors concluded that the autologous scleral implant, although cheaper, is similar to the collagen implant as it stimulates fibrosis, thus decreasing postoperative filtration and leading to a recurrence in raised IOP. This implant caused fibrosis was resolved by Nd:YAG goniopuncture allowing the target IOP to be reached, however the authors concluded that the implant derived from sclera causes more fibrosis as it is a non-viable material [11].

With the Ahmed’s suture the need for gonio-puncture at the end of the 12-month follow-up was only 12% (lower than with both the collagen and scleral implants) and complete success was 81% (higher than with the implants and at a lower IOP cut off than studies using the collagen implant). Historically, nylon is a nonabsorbable monofilament suture composed of polyamides, with minimal induction of cellular response and prolonged tensile strength retention [13].

The proposed Ahmed’s sub-flap mattress suture enhances the IOP-lowering effect of DS via several mechanisms [7], mainly through marinating the intrascleral decompression space and upward elevating of the proximal part of the superficial scleral flap, hence reducing the pressure on the percolating TDM. Also, tangential tightening of the suture to the limbus widens the TDM. An additional advantage of the suture is the gonioscopic visibility of the proximal limb of the suture in the SC (Fig. 1), this aids the visualization of the TDM when YAG-goniopuncture is required [7].

A 2005 study, 3 mm of 0 chromic catgut was used instead of an implant to maintain the aqueous decompression space. The catgut was sutured to the scleral bed with 10/0 nylon, which was also used for closure of the sclera and conjunctiva. Augmentations with antimetabolites was not done [14]. The authors’ reasoning it that absorption of chromic catgut is completely occurs by the 16th day, but the foreign body giant cell reaction is prolonged, continuing despite absorption of the suture (approximately until the 99th day) [15].

Complete success rate, was defined as an IOP lower than 21 mmHg without medication, was achieved in 77% of eyes at 36 months. Qualified success rate, defined as an IOP lower than 21 mmHg with medication, was 100% at 36 months. However, this is an uncontrolled study with a relatively small number of patients (23 eyes) who the authors identified as having “low risk open angle glaucoma” and required post-operative anti-inflammatory treatment for over 5 months [14].

In our surgical technique used mitomycin at a concentration of 0.4 mg/ml Mitomycin-C (MMC)^®^ was used in all cases because of the relatively young age of the participants (39.5 ± 14.8 in group A and 38.3 ± 14.0 in group B) and there is a high incidence of scarring in patients of African origin [16, 17].

Our participants included both primary open angle glaucoma (POAG) and juvenile open angle glaucoma (JOAG) patients. JOAG is often diagnosed at a later stage, with more advanced glaucomatous changes and wider IOP fluctuations than POAG. However, in our study the Ahmed’s subscleral mattress suture was successful in this often-challenging group of patients [18].

The incidence of complications was negligible in both groups, deeming this modification both efficient and safe. The only complication seen was a scleral dehiscence due to trauma which can be attributed to any glaucoma surgery due to the weakness of the surgical site in any DS surgery.

Though the present study evaluated the use of 10/0 Nylon suture, other suture materials like Prolene may also be used. In addition, other modifications such as changing the suture shape to a figure of 8 could achieve the same goals, as long as the suture extends beyond the edges of the superficial flap and is secured tightly.

Drawbacks of our study, lie in the length of the follow-up (12 months vs. up to 36 months follow up in other DS studies). It is randomized and comparative using a control group of conventional DS and the same surgeon in all cases, but we recommend addition of more variables (i.e., comparison of groups by ages, severity of glaucoma).

In conclusion, the Ahmed’s suture, a simple, novel and economic modification to DS, has proven in our study to maintain lower IOP and higher success rates over conventional DS, throughout the 12 months follow-up period.

## Summary

### What was known before

- Deep Sclerectomy is an effective IOP-lowering surgery in open angle glaucoma.
- Deep sclerectomy averts complications seen in penetration surgery.

### What this study adds

- The Ahmed sub-flap mattress suture; act as a hinge maintaining the decompression space to augment the IOP-lowering effect of NPDS.
- The Ahmed’s suture maintains lower IOP levels and has a higher success rate over conventional DS, as it is nearly 30% more effective in reducing the IOP.

## Data Availability

Data are available from authors upon request.

## References

[1] Rulli E, Biagioli E, Riva I, Gambirasio G, De Simone I, Floriani I (2013). Efficacy and safety of trabeculectomy vs nonpenetrating surgical procedures: a systematic review and meta-analysis. JAMA Ophthalmol.

[2] Tiakumzuk S, Mermoud A. Nonpenetrating deep sclerectomy, viscocanalostomy and ancillary goniopuncture. In: Shaarawy T, Dada T, Bhartiya S, editors. ISGS Textbook of Glaucoma Surgery, 1st ed. India:Jaypee; 2014.

[3] Al Obeidan S (2009). Nonpenetrating deep sclerectomy. Expert Rev Ophthalmol.

[4] Varga Z, Shaarawy T (2009). Deep sclerectomy: safety and efficacy. Middle East Afr J Ophthalmol.

[5] Shaarawy T, Nguyen C, Schnyder C, Mermoud A (2004). Comparative study between deep sclerectomy with and without collagen implant: long-term follow-up. Br J Ophthalmol.

[6] Bettin P, Di Matteo F, Rabiolo A, Fiori M, Ciampi C, Bandello F (2016). Deep sclerectomy with mitomycin c and injectable cross-linked hyaluronic acid implant: long-term results. J Glaucoma.

[7] Abdelrahman AM, Habib MM (2020). Sub-flap Mattress Suture with Deep Sclerectomy: A Novel Step. J Glaucoma.

[8] Shaarawy T, Grehan F, Sherwood M. Guidelines on Reporting and Designing of Glaucoma Clinical Trials. Amsterdam, The Netherlands: Kugler Publications; 2008–2009.

[9] Okimoto S, Kiuchi Y, Akita T, Tanaka J (2014). Using the early postoperative intraocular pressure to predict pressure control after a trabeculectomy. J Glaucoma.

[10] Shaarawy T, Flammer J, Smits G, Mermoud A (2004). Low first postoperative day intraocular pressure as a positive prognostic indicator in deep sclerectomy. Br J Ophthalmol.

[11] Devloo S, Deghislage C, Van Malderen L, Goethals M, Zeyen T (2005). Non-penetrating deep sclerectomy without or with autologous scleral implant in open-angle glaucoma; medium-term results. Graefes Arch Clin Exp Ophthalmol.

[12] Shaarawy T, Mansouri K, Schnyder C, Ravinet E, Achache F, Mermoud A (2004). Long-term results of deep sclerectomy with collagen implant. J Cataract Refract Surg.

[13] Salthouse T, Matlaga B, Wykoff M (1977). Comparative tissue response to six suture materials in rabbit cornea, sclera, and ocular muscle. Am J Ophthalmol.

[14] Wevill MT, Meyer D, Van Aswegen E (2005). A pilot study of deep sclerectomy with implantation of chromic suture material as a collagen implant: medium-term results. Eye (Lond).

[15] Lawrie P. Studies in the absorption of surgical catgut. Ph.D Thesis, Edinburgh University Edinburgh:W Blackwood & Sons Ltd; 1955.

[16] Ollikainen M, Puustjärvi T, Rekonen P, Uusitalo H, Terasvirta M (2011). Mitomycin C-augmented deep sclerectomy in primary open-angle glau- coma and exfoliation glaucoma: a three-year prospective study. Acta Ophthalmol.

[17] Cabourne E, Clarke J, Schlottmann P, Evans J (2015). Mitomycin C versus 5-Fluorouracil for wound healing in glaucoma surgery. Cochrane Database Syst Rev.

[18] Kwun Y, Lee E, Han J, Kee C (2016). Clinical Characteristics of Juvenile-onset Open Angle Glaucoma. Korean J Ophthalmol.

